# Olive Leaves for Functional Herbal Infusions: Effects of Post-Harvest Processing on Bioactive Properties, α-Amylase Inhibition Activity and Consumer Safety

**DOI:** 10.3390/molecules31203735

**Published:** 2026-10-18

**Authors:** Liliana Hnatova, Zuzana Knazicka, Eva Kovacikova, Branislav Galik, Katarina Fatrcova Sramkova, Dominika Lenicka, Ivona Janco, Marian Tomka, Olga Grygorieva, Tunde Jurikova, Jiri Mlcek, Marcela Capcarova

**Affiliations:** 1 Institute of Nutrition and Genomics, Faculty of Agrobiology and Food Resources, Slovak University of Agriculture in Nitra, Trieda Andreja Hlinku 2, 949 76 Nitra, Slovakia; xhnatoval@uniag.sk (L.H.); eva.kovacikova@uniag.sk (E.K.); branislav.galik@uniag.sk (B.G.); katarina.sramkova@uniag.sk (K.F.S.); dominika.lenicka@uniag.sk (D.L.); 2 AgroBioTech Research Centre, Slovak University of Agriculture in Nitra, Trieda Andreja Hlinku 2, 949 76 Nitra, Slovakia; ivona.janco@uniag.sk; 3 Institute of Biotechnology, Faculty of Biotechnology and Food Sciences, Slovak University of Agriculture in Nitra, Trieda Andreja Hlinku 2, 949 76 Nitra, Slovakia; marian.tomka@uniag.sk; 4 M. M. Gryshko National Botanical Garden, National Academy of Sciences of Ukraine, Sadovo-Botanichna 1, 010 14 Kyiv, Ukraine; olgrygorieva@gmail.com; 5 Institute for Teacher Training, Faculty of Central European Studies, Constantine the Philosopher University in Nitra, Drazovska 4, 949 74 Nitra, Slovakia; 6 Department of Food Analysis and Chemistry, Faculty of Technology, Tomas Bata University in Zlin, Vavreckova 5669, 760 01 Zlin, Czech Republic; mlcek@utb.cz; 7 Institute of Applied Biology, Faculty of Biotechnology and Food Sciences, Slovak University of Agriculture in Nitra, Trieda Andreja Hlinku 2, 949 76 Nitra, Slovakia; marcela.capcarova@uniag.sk

**Keywords:** *Olea europaea* L., leaves, herbal infusions, post-harvest processing, biological properties, mineral composition, health risk assessment

## Abstract

Olive leaves (OL; *Olea europaea* L.) are an abundant by-product of olive production with functional and nutraceutical potential. This study evaluated the effects of post-harvest treatments using fresh, air-dried, and lyophilized OL of cv. ‘Frantoio’, which were infused in water at 85 °C for 10 min. The antioxidant capacity (AC; DPPH method), total phenolic content (TPC; Folin–Ciocalteu assay), and α-amylase inhibition activity of the herbal infusions were assessed. Macro- and trace elements were determined directly in air-dried and lyophilized OL by inductively coupled plasma optical emission spectrometry (ICP-OES). Potential non-carcinogenic health risks were assessed using estimated daily intake (EDI), target hazard quotient (THQ), and total THQ (TTHQ). Lyophilized OL herbal infusions exhibited the highest AC (15.32 ± 0.85%), TPC (65.61 ± 1.15 mg GAE/g DW), and α-amylase inhibition activity (23.34%). The mineral analysis identified Ca and K as the predominant macroelements, whereas Cd and Pb were below the analytical limit of detection. All individual THQ and cumulative TTHQ values remained below 1, indicating no appreciable non-carcinogenic health risk under the modeled exposure conditions. These results identify lyophilization as a favorable processing option for preserving the functional potential of OL and support their potential use as raw material for functional infusions and other food applications.

## 1. Introduction

The Mediterranean dietary pattern is characterized by a high contribution of plant-derived foods and beverages, including vegetables, fruits, herbs, olives, olive oil, and plant infusions. These foods provide numerous compounds with biological activity, such as phenolics, flavonoids, vitamins, and minerals [1,2]. Their regular consumption has been linked to favorable effects on cardiometabolic health and a lower risk of several chronic disorders, including *diabetes mellitus*, cardiovascular disease, cancer, and neurodegenerative conditions; beneficial effects on intestinal health have also been described [1,3,4].

Among the characteristic components of the Mediterranean diet, olives and olive oil hold a particularly important position. The olive tree (*Olea europaea* L.) is therefore one of the most typical and economically significant crops in Mediterranean countries, where it is mainly cultivated for olive oil production [5,6,7]. However, this industry also generates large amounts of by-products, including olive leaves (OL), which are produced during harvesting, pruning, and olive processing. Olive leaves may represent approximately 5–10% of the mass of olives entering a mill, yet this biomass is frequently discarded or used only to a limited extent [8,9,10]. Valorization of this material is, therefore, of interest from both sustainability and food-development perspectives. Olive leaves are an inexpensive, renewable, abundant, and valuable source of health-promoting compounds, thereby attracting increasing interest in their use across several fields, including the food, nutraceutical, pharmaceutical, and cosmetic industries [11,12]. The functional interest in OL is largely associated with their phytochemical profile. They contain several important phenolic and secoiridoid compounds, including oleuropein, hydroxytyrosol, tyrosol, verbascoside, and ligstroside [11,13]. These constituents have been associated with antioxidant, anti-inflammatory, antimicrobial, antihypertensive, hypocholesterolemic, and antidiabetic effects [14,15,16].

Traditional consumption of OL as herbal infusions further supports their potential use in beverage formulations. Consumer interest in plant-based infusions has expanded beyond conventional tea to a broad range of herbal materials [17], but OL remain comparatively underutilized despite their availability and phytochemical value. Previous preliminary studies have applied different conditions for the preparation of OL infusions. Ramírez et al. [17] prepared OL tea using dried leaves in 240 mL of boiling water for 5 min, while Zafar et al. [18] used OL powder in 250 mL of hot water with an infusion time of 7–10 min. Preczenhak et al. [19] demonstrated that infusion conditions substantially influence the extraction of phenolic compounds and antioxidant capacity (AC), applying a temperature of 80 °C for 10 min, whereas Meireles et al. [20] prepared OL infusions from commercially available dried OL according to the manufacturer’s instructions using 1.0 g of chopped leaves in 250 mL of water at 90 °C for 5 min. Accordingly, in the present study, aqueous infusions of cv. ‘Frantoio’ OL were prepared under standardized conditions using an amount of fresh, air-dried, or lyophilized material equivalent to 1.70 g of dry matter in 250 mL of water at 85 °C for 10 min.

For food applications, the post-harvest condition of the leaves is particularly important. Enzymatic reactions may continue after collection, and polyphenol oxidases and related enzymes can promote oxidation and transformation of phenolic constituents unless the material is rapidly stabilized [21]. Dehydration is one of the principal strategies used to reduce water activity and limit these reactions. Air-drying is inexpensive and technically simple, whereas lyophilization is more time- and energy-intensive but is generally associated with improved preservation of thermolabile plant constituents [22]. Previous studies have indicated that both approaches can retain OL phenolics, although the final composition depends on the specific drying conditions [23,24,25]. Although the effects of drying on OL antioxidant properties and phenolic profiles have been studied extensively [9,25,26,27], direct comparisons of fresh, air-dried, and lyophilized leaves prepared specifically as aqueous herbal infusions for human consumption remain scarce. The majority of previous studies have focused on dried leaf material or solvent-based extracts rather than on a side-by-side evaluation of differently processed OL under standardized aqueous infusion conditions. Consequently, it remains unclear the extent to which post-harvest processing influences the aqueous extractability of bioactive compounds, resulting in functional properties of the final infusion. The present study fills this gap by directly comparing fresh, air-dried, and lyophilized OL of the same cultivar under identical infusion conditions.

Beyond their antioxidant properties, OL are also of interest in relation to carbohydrate digestion. α-Amylase catalyzes the hydrolysis of dietary starch, and partial inhibition of this enzyme may slow carbohydrate breakdown and attenuate the postprandial glycemic response [28]. Because phenolic compounds may interact with digestive enzymes through hydrogen bonding, hydrophobic interactions, and other non-covalent mechanisms, OL have been investigated as a source of naturally occurring constituents with potential antidiabetic activity [29].

The evaluation of mineral composition is important for determining the nutritional value and safety of OL, particularly when considering their use in functional beverages or as nutraceutical ingredients. The mineral matter profile of OL may vary considerably depending on cultivar, soil properties, climate, geographical origin, and agronomic management [30,31,32]. Calcium (Ca) and potassium (K) are commonly reported among the most abundant elements in OL, together with lower concentrations of sodium (Na), magnesium (Mg), phosphorus (P), manganese (Mn), iron (Fe), copper (Cu), zinc (Zn), and other trace elements [33,34]. Calcium contributes to skeletal maintenance and several cellular processes, while K is involved in electrolyte homeostasis, neuromuscular function, and the regulation of cardiac activity [35,36,37]. Adequate intake of these macroelements is also relevant to the prevention of cardiovascular diseases [38]. However, in addition to nutritionally relevant elements, the presence of trace and potentially toxic elements should also be taken into account when evaluating OL intended for human consumption. Therefore, assessment of their mineral matter composition should be accompanied by a clear definition of the exposure assumptions used for health-risk characterization.

To ensure transparency of the subsequent health-risk assessment, the exposure model used in the present study was based on a daily OL intake of 10 g/person/day [39] and a default adult body weight (BW) of 70 kg according to EFSA guidance [40]. The assessment was based on total elemental concentrations measured directly in the air-dried and lyophilized leaf material. Consequently, the resulting estimated daily intake (EDI), target hazard quotient (THQ), and total target hazard quotient (TTHQ) values represent a modeled leaf-based exposure scenario rather than the actual elemental intake from the prepared infusion.

Given this background, the present study investigated the effects of post-harvest processing on the functional properties and elemental safety of OL, with particular attention to the direct comparison of fresh, air-dried, and lyophilized leaves under standardized aqueous infusion conditions. We hypothesized that post-harvest processing can significantly influence the functional properties of OL infusions, with lyophilization providing better preservation and aqueous extractability of bioactive constituents than air-drying. In parallel, the mineral matter composition and associated non-carcinogenic health risk were evaluated directly in air-dried and lyophilized leaf material to provide complementary information on the nutritional and safety aspects of processed OL. The specific objectives were: (i) to compare AC and total phenolic content (TPC) in herbal infusions prepared from fresh, air-dried, and lyophilized leaves; (ii) to determine the α-amylase inhibition activity of these infusions; (iii) to characterize differences in the mineral composition of air-dried and lyophilized OL; and (iv) to calculate EDI, THQ, and TTHQ from the measured elemental concentrations to assess potential health risks under the modeled exposure scenario.

## 2. Results and Discussion

### 2.1. Effect of Post-Harvest Processing on the AC and TPC of OL Herbal Infusions

The bioactive potential of OL used for infusion-based products is largely determined by post-harvest and technological processing conditions. The preparation of OL herbal tea typically includes washing, drying, grinding, and packaging, with drying being one of the most relevant technological steps. This phase can substantially affect the concentration, stability, and extractability of phenolic and other bioactive compounds in the final product. As has been reported previously, pre-treatments applied to leaves before infusion may significantly influence the amount of target compounds released into the beverage [17,41]. Moreover, drying-induced disruption of plant tissues may facilitate interactions between phenolic compounds and endogenous enzymes, thereby contributing to their transformation or degradation during processing [42,43].

**Figure 1:**
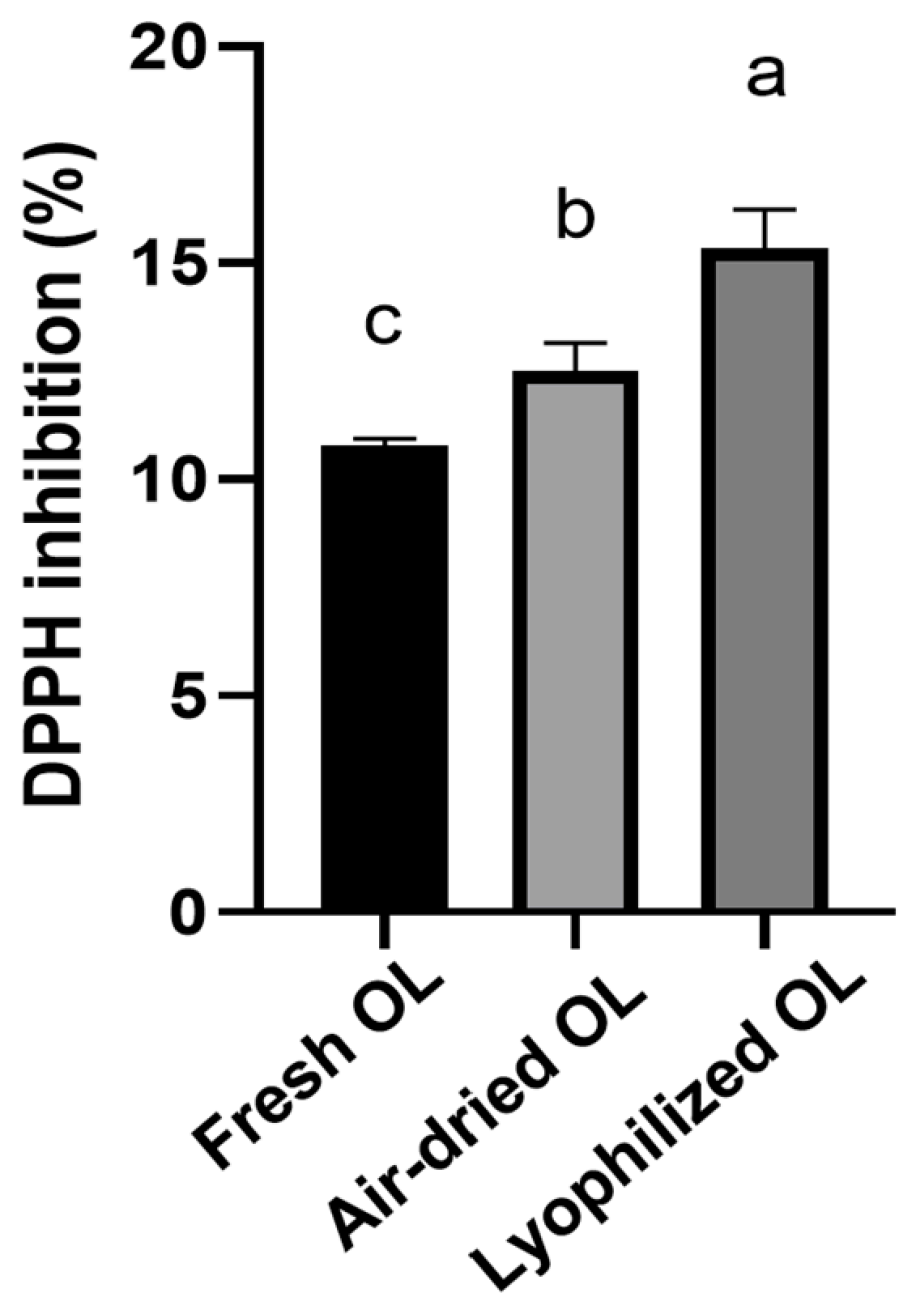
Antioxidant capacity of infusions prepared from fresh, air-dried, and lyophilized OL. Values are presented as mean ± standard deviation; different lowercase letters within the same column indicate statistically significant differences among samples (One-way ANOVA with Tukey’s multiple-comparisons test; α = 0.05).

In the present study, the herbal infusion prepared from lyophilized OL exhibited the highest AC (15.32 ± 0.85%), followed by air-dried (12.49 ± 0.60%) and fresh leaves (10.76 ± 0.15%; *p* < 0.05). The results are presented in Figure 1. Relative to the fresh-leaf infusion, AC increased by approximately 16.08% in the air-dried treatment and 42.38% in the lyophilized treatment. Among the evaluated processing methods, lyophilization appeared to be the most effective approach for preserving the extractability of compounds contributing to the AC of OL infusions. The importance of processing and preparation conditions for OL tea quality was also demonstrated by Preczenhak et al. [19], who investigated the effect of the preparation method on the availability of polyphenols and AC in OL tea prepared from dried OL of the ‘Arbequina’ cultivar. Their study showed that the preparation method significantly affected the release of bioactive compounds into the tea infusion. In particular, boiling enhanced the release of phenolic acids and oleuropein, thereby increasing TPC and AC. These findings support the assumption that the antioxidant potential of OL tea infusions is strongly dependent not only on the initial post-harvest treatment of OL but also on the subsequent preparation procedure.

The phenolic content and AC of OL are also influenced by cultivar, olive tree age, cultivation area, and sampling time associated with the phenological stage [31,44,45,46,47,48,49]. Although studies specifically focused on ‘Frantoio’ OL tea infusions are limited, Orak et al. [50] investigated lyophilized aqueous OL extracts from 21 cultivars, including ‘Frantoio’, and demonstrated their notable antioxidant properties using several in vitro assays. Interestingly, ‘Frantoio’ exhibited the highest total flavonoid content among the evaluated cultivars, suggesting that this cultivar may represent a valuable source of water-extractable antioxidant compounds. Moreover, Monteleone et al. [16] proposed an organic solvent-free, water-based extraction procedure for obtaining oleuropein-rich OL extracts, supporting the use of aqueous OL preparations for functional beverage applications. This approach is particularly important because water-based extraction avoids the use of potentially toxic organic solvents and is therefore more suitable for food-grade products intended for human consumption. Similar aqueous extraction strategies using boiling water have also been applied to recover phenolic compounds from plant materials, including *Salvia triloba* L. leaves, *Tilia argentea* flowers, green and black tea leaves, and grapes [51,52].

The trend observed in the present study is consistent with our previously published investigation, which used the same type of plant material but employed acidified hydromethanolic extraction (80% methanol, 18% water, and 2% acetic acid). In that study, lyophilized OL exhibited the highest AC (70.12 ± 0.64%). Compared with fresh OL, AC increased by approximately 37.70% in air-dried and 38.20% in lyophilized samples [53]. Castillo-Luna et al. [54] confirmed that drying conditions modified both the qualitative and quantitative phenolic profiles of extracts obtained from olive-tree materials, including leaves, fruits, and pomace. Zhang et al. [27] further reported that freeze-drying and hot-air drying differentially affected the phytochemical profile and biological activity of OL, indicating that preservation of antioxidant potential depends not only on TPC but also on the specific compound classes retained during processing. Using UPLC-Q-Exactive Orbitrap-MS, the authors identified 33 bioactive compounds and demonstrated that freeze-dried OL contained higher levels of several flavonoid aglycones, including luteolin, quercetin, kaempferol, apigenin, hispidulin, eriodictyol, and taxifolin, as well as hydroxytyrosol, whereas hot-air-dried samples contained higher levels of the iridoids (e.g., oleuropein and secoxyloganin). These findings indicate that individual phenolic classes respond differently to drying conditions and that lyophilization does not necessarily maximize the retention of all phenolic compounds. Similar compound-specific effects have been reported in other phytochemical profiling studies. Ahmad-Qasem et al. [55] showed that freeze-drying and hot-air drying produced distinct phenolic profiles in OL (var. Serrana) and that drying conditions significantly influenced the concentrations of individual polyphenols. More recently, Chaji et al. [56] compared air-drying, oven-drying, freeze-drying, and solar-drying in four olive cultivars and demonstrated qualitative and quantitative differences in their phenolic profiles depending on both the drying method and cultivar. In particular, the drying method associated with the highest oleuropein content differed among cultivars, further emphasizing that the optimal processing approach may depend on both the plant material and the target bioactive compounds.

The superior performance of the lyophilized samples in the present study may therefore reflect reduced oxidation and enzymatic degradation of phenolic constituents during dehydration. Lyophilization minimizes thermal exposure and limits conditions that may promote oxidative and enzymatic changes, which can be particularly important for thermolabile flavonoids and hydroxytyrosol-related compounds. Compared with high-temperature drying, several non-glycosylated flavonoids are more susceptible to thermal degradation, whereas some iridoids, including oleuropein, may show greater thermal stability [27]. In the present study, however, the comparison involved air-drying at room temperature rather than high-temperature hot-air drying; therefore, the observed advantage of lyophilization should not be attributed exclusively to reduced thermal degradation. Reduced exposure to oxygen and enzymatic reactions during the drying process, together with structural changes induced by freeze-drying, may also contribute to improved preservation and subsequent aqueous extractability of phenolic constituents.

By minimizing thermal stress, lyophilization may preserve thermolabile molecules and generate a porous matrix that facilitates their subsequent extraction into water. Nevertheless, because individual phenolics were not quantified, the contribution of oleuropein, hydroxytyrosol derivatives, verbascoside, or flavonoids cannot be assigned directly. Thus, the higher AC observed in the lyophilized OL infusions may reflect not only improved overall preservation and extractability of phenolic compounds but also differences in the qualitative phytochemical profile resulting from the drying process. These findings demonstrate that processing conditions should be optimized to maximize the retention and aqueous extractability of bioactive compounds in OL intended for infusion-based products.

**Figure 2:**
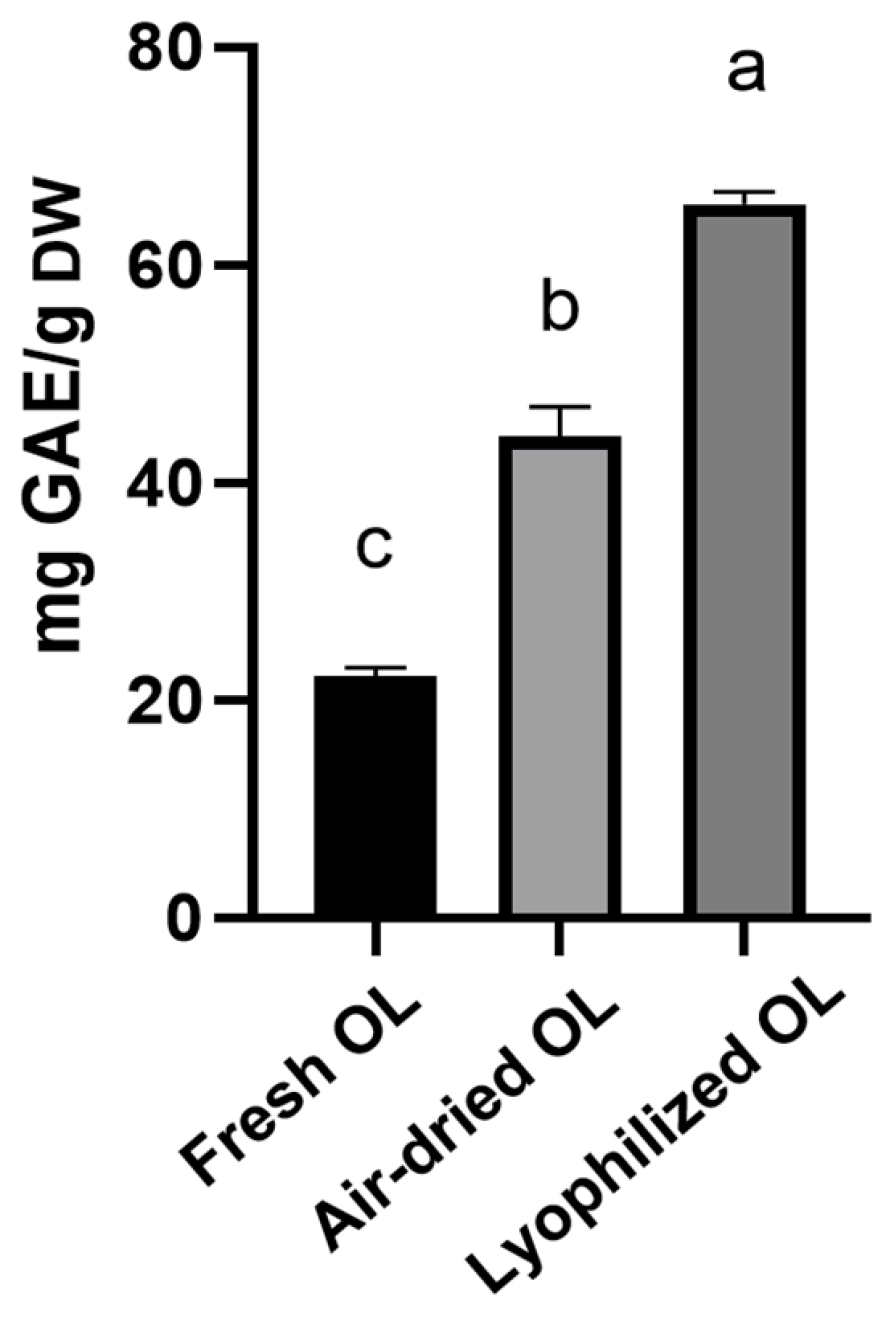
Total phenolic content of infusions prepared from fresh, air-dried, and lyophilized OL. Values are presented as mean ± standard deviation; different lowercase letters within the same column indicate statistically significant differences among samples (One-way ANOVA with Tukey’s multiple-comparisons test; α = 0.05).

As shown in Figure 2, TPC in differently processed OL herbal infusions ranged from 22.22 to 65.61 mg gallic acid equivalent (GAE)/g dry weight (DW). The herbal infusion prepared from lyophilized OL exhibited significantly (*p* < 0.05) higher TPC levels (65.61 ± 1.15 mg GAE/g DW) than those of air-dried (44.27 ± 2.77 mg GAE/g DW) and fresh OL samples (22.22 ± 0.84 mg GAE/g DW). These findings indicate that lyophilization markedly improved the release of phenolic compounds into the infusion. Compared with the fresh OL infusion, TPC increased by approximately 195.30% in the lyophilized sample, whereas AC increased by approximately 42.40%. Thus, the large increase in TPC was accompanied by a comparatively smaller increase in AC. This discrepancy suggests that the DPPH response depends not only on the total amount of extracted phenolics but also on the chemical structure, reactivity, and interactions with other compounds present in the infusion. The Folin–Ciocalteu method used for TPC quantification broadly detects reducing substances, whereas the DPPH assay reflects the ability of constituents to neutralize free radicals. Therefore, an increase in TPC does not necessarily correspond to a proportionally higher AC [57]. The increased TPC observed in lyophilized OL may be related to improved extractability and recovery of phenolics caused by structural changes in plant tissues during lyophilization. Such changes can facilitate the release of bound phenolic compounds from disrupted cellular structures, although this method is generally limited by its long processing time and relatively high cost [22,58]. This is consistent with previous studies showing that drying and grinding are critical processing steps for OL intended for infusion purposes, as they influence the retention and diffusion of bioactive compounds into the final beverage [17]. Similarly, Filgueira-Garro et al. [9] reported that freeze-drying and air-drying were among the most suitable drying methods for preserving TPC and AC in OL. The higher TPC observed in the lyophilized samples of the present study is consistent with the capacity of freeze-drying to preserve sensitive constituents and to promote their subsequent extractability, although, as discussed above, the response of individual phenolic compounds may differ according to their chemical class, cultivar, and processing conditions [27,55,56]. This method is widely used for the stabilization of high-quality foods, biological materials, and pharmaceuticals because it helps preserve sensitive compounds during dehydration [59,60].

Beyond their use in herbal infusions, processed OL may represent a valuable raw material for standardized powders, functional food ingredients, nutraceutical extracts, and dietary supplements, as demonstrated by their incorporation into bakery products and fermented dairy matrices in previous studies [61,62,63,64]. This potential is further supported by advances in food-compatible extraction technologies aimed at recovering phenolic-rich fractions, particularly oleuropein and related compounds [12,16]. Clodoveo et al. [12] highlighted innovative extraction technologies for the development of OL-derived functional ingredients, further supporting the broader valorization of this plant material. However, practical translation to larger-scale production requires consideration of processing efficiency, product stability, energy demand, and economic feasibility. Although lyophilization provided the most favorable functional properties under the conditions of the present study, this technique is generally associated with relatively long processing times and high operating costs, which may limit its routine industrial application [22,27]. In contrast, hot-air drying is more widely used on an industrial scale and may represent a more feasible alternative when processing conditions are optimized to preserve the desired phytochemical profile [27]. Therefore, future studies should be focused on both the identification and quantification of individual phenolic compounds responsible for the observed responses and the technological scale-up of OL processing. In particular, lyophilization should be compared with controlled convective and other alternative drying technologies in terms of bioactive compound retention, processing time, energy consumption, and economic feasibility.

In addition to phytochemical retention and processing feasibility, sensory acceptability and storage stability are important considerations for the practical application of OL-derived infusion ingredients. Tripodi et al. [65] demonstrated that drying conditions influenced both the volatile composition and sensory properties of OL herbal tea, including bitterness, astringency, and consumer acceptance, with air-dried infusions showing a more favorable sensory profile than microwave-dried samples under the conditions evaluated. Similarly, Vieitez et al. [66] reported that reducing OL particle size increased the extraction of polyphenols and oleuropein; however, infusions prepared from powdered leaves exhibited more intense bitterness, astringency, and green sensory attributes, which negatively affected consumer acceptability. These findings indicate that maximizing the extraction of bioactive compounds does not necessarily result in optimal sensory quality. Ahmad-Qasem et al. [25] further demonstrated that OL drying and extract dehydration influenced the initial phenolic profile and its evolution during storage, highlighting the relevance of storage stability in the development of OL-derived products. Although sensory evaluation was not within the scope of the present study, these aspects should be considered in future product-development studies as part of the translational pathway for OL-derived infusion ingredients, together with technological scalability and economic feasibility.

### 2.2. Effect of Post-Harvest Processing on the α-Amylase Inhibition Activity of OL Herbal Infusions

Individual phenolic compounds and specific phenolic classes may influence digestive-enzyme activity independently of their antioxidant properties [67]. α-Amylase is a key carbohydrate-hydrolyzing enzyme involved in starch digestion, and its inhibition may contribute to slower postprandial glucose release. In the present study, α-amylase inhibition activity differed according to the post-harvest treatment (Table 1), ranging from 6.91 to 23.34%. The infusion prepared from lyophilized OL showed the highest α-amylase inhibition (23.34%; *p* < 0.05), followed by infusions prepared from fresh (10.84%) and air-dried OL (6.91%). The absolute difference was 12.50 percentage points between the lyophilized and fresh treatments and 16.43 percentage points between the lyophilized and air-dried treatments. Thus, constituents extracted from lyophilized OL were more effective in reducing α-amylase activity under the applied in vitro conditions. These results indicate α-amylase inhibitory potential under in vitro conditions and should therefore be regarded as a preliminary screening of biological activity rather than evidence of antidiabetic efficacy.

Previous research supports the presence of α-amylase-modulating constituents in OL. Al-Dabbas et al. [68] reported that an aqueous infusion of wild OL displayed both α-amylase and α-glucosidase inhibition activities, while Komaki et al. [29] identified luteolin glucosides and oleanolic acid among the compounds contributing to the inhibition of human salivary and pancreatic α-amylases. Phenolic compounds may reduce catalytic activity by interacting with amino acid residues within or near the enzyme active site through hydrogen bonding and hydrophobic interactions [69,70]. The stronger inhibition observed for the lyophilized samples may therefore be associated, at least partly, with improved preservation and aqueous extraction of enzyme-interacting constituents. However, the present data do not demonstrate a direct causal relationship between TPC and α-amylase inhibition, because TPC is a non-specific measure and the study did not quantify individual compounds or formally test correlations among TPC, AC, and enzyme inhibition. Consequently, OL-derived infusions should be regarded as promising matrices for further mechanistic investigation rather than as clinically established modulators of postprandial glycemia.

Comparable α-amylase inhibitory effects have also been reported for other tea and plant-based beverages rich in phenolic compounds. Striegel et al. [71] showed that black tea water extracts and polyphenol-enriched black tea fractions inhibited carbohydrate-hydrolyzing enzymes, with the activity depending partly on the composition of phenolic fractions, including high-molecular-weight phenolic compounds. Yilmazer-Musa et al. [72] also reported that grape seed, green tea, and white tea extracts inhibited α-amylase and α-glucosidase activities, although their inhibitory potency differed according to the type and structure of the polyphenols present. These findings support the assumption that α-amylase inhibition by plant-derived infusions is not determined only by total phenolic content but also by the specific phenolic profile and the presence of compounds with higher affinity for digestive enzymes. Direct numerical comparison with other studies is limited by differences in assay conditions, extract preparation, and extract concentrations.

Importantly, the α-amylase inhibition observed in the present study represents an in vitro screening result and should be distinguished from the outcomes of the human intervention studies discussed below, which assessed physiological glycemic and metabolic responses. The possible relevance of OL tea to postprandial glycemic regulation has also been examined in human interventions, although outcomes are not uniform. Wainstein et al. [73] reported improvements in hemoglobin A1c (HbA1c) and fasting immunoreactive insulin (IRI) levels in patients with type 2 diabetes after daily treatment with 500 mg of OL extract for 14 weeks; however, postprandial IRI levels did not change. Araki et al. [74] explored the health benefits of OL tea in Japanese adults with prediabetes who were mildly obese or had a normal-high body mass index. In their 12-week intervention, participants consumed 330 mL of the test beverage three times daily with meals. OL tea consumption was associated with a decrease in fasting plasma glucose (FPG), whereas insulin levels and HbA1c remained unchanged. The results also suggested a suppressive effect of OL tea on blood lipid elevation. Similarly, an earlier study by Araki et al. [75] showed that regular OL tea intake led to a significant decrease in waist circumference and FPG. Meireles et al. [20] evaluated whether OL tea could represent a dietary strategy for improving the glycemic response after ingestion of a carbohydrate-rich meal. However, their results did not fully support this hypothesis, as OL tea did not improve the postprandial glycemic curve induced by the meal. Nevertheless, the authors observed a delay in the glycemic peak after OL tea consumption, suggesting that this effect should be further explored. They also emphasized the need for future studies evaluating chronic OL tea consumption, including its potential impact on HbA1c and other metabolic biomarkers involved in long-term glycemic regulation. More recently, Potsaki et al. [63] contributed to the growing body of evidence examining the role of OL supplementation in metabolic health, particularly with respect to postprandial responses in healthy adults. They evaluated OL tea and OL powder biscuits in a randomized controlled crossover trial and observed a delayed insulin response after OL interventions, although no significant changes were found in the primary glycemic and lipid biomarkers.

Several limitations of the present study should be acknowledged. The investigation was limited to a single olive cultivar (cv. ‘Frantoio’), and the drying procedures and infusion preparation were conducted under laboratory-scale conditions; therefore, the findings may not be directly generalizable to other cultivars or transferable to pilot- or industrial-scale processing. In addition, α-amylase inhibition was evaluated exclusively in vitro. The results indicate that OL herbal infusions contain constituents capable of inhibiting α-amylase activity. Their physiological and nutritional relevance, however, requires further confirmation through detailed phytochemical profiling, simulated gastrointestinal digestion, in vivo models, and appropriately designed human intervention studies. This need is further supported by recent reviews highlighting that the biological effects of major olive phenolics depend not only on their concentration but also on their gastrointestinal transformation, absorption, metabolism, and resulting bioavailability. Nikou et al. [76] emphasized substantial knowledge gaps in the metabolism and bioavailability of hydroxytyrosol, tyrosol, oleuropein, and related secoiridoids across in vitro, animal, and human studies. Jordán et al. [77] further highlighted the importance of the food matrix, formulation, metabolic transformation, and gut microbiota in determining hydroxytyrosol bioavailability, while Khalil et al. [78] reviewed the absorption, metabolism, and bioavailability of oleuropein and its relevance to nutraceutical applications.

### 2.3. Mineral Analysis of Air-Dried and Lyophilized OL and Human Health Risk Assessment

The mineral matter analysis of *Olea europaea* leaves (cv. ‘Frantoio’) subjected to different post-harvest treatments included 18 elements, while the potentially toxic elements (Cd and Pb) were below the limit of detection (<LOD) of the inductively coupled plasma optical emission spectrometry (ICP-OES) method used (Table 2). The overall distribution of the quantified elements in air-dried OL was as follows (mg/kg DW): K > Ca > Na > Mg > aluminum (Al) > strontium (Sr) > Fe > Mn > Zn > barium (Ba) > Cu > selenium (Se) > nickel (Ni) > chromium (Cr) > lithium (Li) > silver (Ag). The lyophilized OL samples exhibited a similar composition, with Ca, K, Na, and Mg representing the most abundant macroelements. The dominance of K and Ca is consistent with previous studies conducted across various OL cultivars and production regions [34,35,53,79,80], which identified these elements as the major macroelements in OL. These findings further support the potential of OL, an abundant agricultural by-product, as a source of minerals for nutraceutical and functional food applications [34]. Both macroelements are particularly relevant because of their involvement in numerous physiological and metabolic processes in the human body. Calcium contributes primarily to skeletal mineralization, muscle contraction, and cellular signaling, whereas K is essential for electrolyte homeostasis, neuromuscular function, and blood-pressure regulation [36,38].

Tunç et al. [81] analyzed dried ‘Frantoio’ leaves by ICP-OES and identified Ca (4897.68 mg/kg) as the predominant element, which is in agreement with our findings. In the present study, the lyophilized OL samples exhibited a significantly (*p* < 0.001) higher Ca concentration than the air-dried OL samples (6931.89 vs. 6054.29 mg/kg DW), whereas K concentration was significantly lower (6448.58 vs. 7467.24 mg/kg DW; *p* < 0.001). These differences may reflect matrix-related effects associated with dehydration and the natural biological heterogeneity of the leaf material. Therefore, the observed differences in elemental concentrations between air-dried and lyophilized OL should be interpreted as differences between the analyzed sample matrices rather than as a direct consequence of the lyophilization process.

From a safety perspective, Cd and Pb were below the detection limit in both air-dried and lyophilized OL in the present study. This finding is consistent with previous investigations reporting very low or non-detectable concentrations of these potentially toxic elements in OL. Pashtetskaia et al. [34] reported Cd and Pb below the detection limits in OL and their corresponding aqueous tisanes, supporting a favorable elemental safety profile of both the raw material and the resulting beverage. Similarly, Pucci et al. [82] reported Pb and Cd below the detection limits in OL samples collected from different Italian production areas. Our results therefore support a favorable elemental safety profile of the analyzed ‘Frantoio’ OL samples. Nevertheless, the accumulation of potentially toxic elements in OL may depend on cultivar, geographical origin, leaf age and physiological status, soil characteristics, and environmental exposure [34,81,82]; therefore, these findings should be interpreted within the specific conditions of the investigated plant material.

Among the trace elements, lyophilized leaves contained significantly (*p* < 0.001) higher concentrations of Al, Sr, Fe, Zn, Ba, Ni, and Li (Table 2). Pucci et al. [82] demonstrated that mineral composition can vary considerably even within a single olive cultivar grown at different Italian sites. For ‘Frantoio’ leaves from Allumiere, Al concentrations ranged from 36 to 84 mg/kg DW, while 69 mg/kg DW was reported for material from the ENEA production area. These values were comparable to those determined in the present study (65.53–86.43 mg/kg DW), whereas the reported Sr, Fe, Zn, Ba, and Cu values were generally higher. Comparable concentrations of several trace elements were also documented by Tunç et al. [81] for dried ‘Frantoio’ OL analyzed using ICP-OES, including Mn (10.77 mg/kg), Cu (2.41 mg/kg), Ni (0.32 mg/kg), and Zn (2.86 mg/kg). Cavalheiro et al. [83] examined several OL cultivars grown in southern Brazil using ICP-OES and reported that Al, Ca, Fe, K, Mg, P, and sulfur (S) were present at the highest concentrations. When expressed in relation to the recommended daily intake, the Fe content calculated for 50 g of dried leaves corresponded to the recommended daily level, whereas the Cu content exceeded the recommended value in all investigated cultivars. The reported results suggest that OL warrant further evaluation as potential food supplements to enhance the nutritional value of the resulting products.

The elemental concentrations determined directly in the leaves can be used as a basis for estimating potential dietary exposure and associated health risks [84,85]. In the present study, EDI, THQ, and TTHQ were calculated from the total elemental concentrations measured directly in air-dried and lyophilized OL. These calculations therefore represent a modeled leaf-based exposure scenario and do not reflect experimentally measured elemental transfer into the prepared infusions. The EDI values calculated for the analyzed elements in OL samples are presented in Table 3. Among the macroelements, K accounted for the highest modeled daily exposure in air-dried OL (1.066748 mg/kg BW/day), whereas Ca showed the highest EDI in lyophilized OL (0.990270 mg/kg BW/day). Our findings reflect the predominance of K and Ca in the mineral profiles of the respective samples (Table 2). For the trace elements, Al had the highest EDI, with values of 0.009361 and 0.012347 mg/kg BW/day for air-dried and lyophilized OL, respectively, followed by Sr and Fe. The monitored trace elements showed generally low EDI values, which remained below the corresponding United States Environmental Protection Agency (US EPA) [86] oral reference doses (RfDs).

The actual exposure to individual elements may vary depending on the form in which OL are consumed. Brima and Siddeeg [87], who investigated hot-water infusions prepared from several medicinal plants, including *Olea europaea* leaves, reported that elemental exposure associated with the consumption of OL infusions remained below the corresponding dietary reference values. More recently, Pashtetskaia et al. [34] demonstrated that the transfer of minerals from OL into tisanes was element-dependent. Macroelements, particularly K, exhibited considerably higher extractability than most trace elements, whereas Al, Ba, lead (Pb), cadmium (Cd), vanadium (V), and Cr were below the analytical detection limits in the resulting infusions.

Long-term consumption of medicinal plants without prior quantitative analysis may lead to exposure to potentially toxic elements, their bioaccumulation in the human body, and consequent adverse health effects. Tefera et al. [85] emphasized the importance of regular elemental monitoring and quantitative health risk assessment of medicinal plant materials intended for prolonged consumption. In their study, the individual THQ values and the cumulative hazard index calculated for *Olea europaea* leaves remained below 1, indicating a low potential for non-carcinogenic health effects under the evaluated exposure conditions. The THQ and TTHQ values calculated for the air-dried and lyophilized OL samples in the present study are shown in Table 4. The individual THQ values for air-dried OL decreased in the following order: Se > Mn > Al > Cu > Cr > Sr > Fe > Li > Ni > Ba > Zn > Ag. For lyophilized OL, the corresponding order was as follows: Al > Mn > Ni > Sr > Cu > Cr > Fe > Li > Se > Ba > Zn > Ag. All individual THQ values were below the threshold of 1 (Table 4), indicating a low probability of adverse non-carcinogenic effects under the modeled exposure assumptions. Among the monitored elements detected in air-dried OL, Se exhibited the highest individual THQ value (0.037000). Nevertheless, this value remained well below the risk threshold, suggesting that long-term consumption of air-dried OL at the estimated intake level is unlikely to result in adverse non-carcinogenic health effects. In lyophilized OL, Al exhibited the highest individual THQ value (0.012347). Its contribution to the overall risk was low, and its THQ value remained substantially below the threshold of concern, suggesting a low potential for adverse non-carcinogenic effects at the estimated exposure level. The low TTHQ values also suggest that combined exposure to the assessed elements through OL consumption is unlikely to pose an appreciable non-carcinogenic health risk. These findings support a low level of concern for the assessed non-carcinogenic elemental risks under the modeled intake scenario; however, they should not be interpreted as a comprehensive demonstration of product safety, because elemental transfer into the prepared infusions and other possible hazards were not evaluated.

## 3. Materials and Methods

### 3.1. Plant Material and Post-Harvest Processing

Fresh leaves of *Olea europaea* L. cv. ‘Frantoio’ were collected from three trees located in the Padova region of Italy, with each tree representing an independent biological replicate. Sampling was conducted in May of each year from 2023 to 2025, during phenological stages corresponding approximately to the early spring development of olive trees, as described by the BBCH (Biologische Bundesanstalt, Bundessortenamt and Chemische Industrie) scale adapted for *Olea europaea* L. Botanical identification and characterization of the plant material were performed in collaboration with the M. M. Gryshko National Botanical Garden (National Academy of Sciences of Ukraine, Kyiv, Ukraine). Leaves were randomly collected from accessible positions throughout the tree canopy to account for variations in sun exposure. After harvest, the plant material was placed in plastic bags, transported to the laboratory, and washed with cold water to remove visible surface contaminants. The fresh OL were then randomly divided into three groups. The first group was stored in vacuum-sealed bags at −80 °C. The second group was air-dried on a bench under ambient laboratory conditions (22–25 °C) for 10 days, until constant weight was reached. The remaining leaves were lyophilized under vacuum (0.50 mbar) for 48 h using a LyoQuest Plus-55 ECO lyophilizer (Telstar, Terrassa, Spain), with temperature maintained at −48 ± 2 °C. After processing, the OL samples were finely ground using a Philips ProBlend 4 blender (model HR2100/40; Philips, Amsterdam, The Netherlands) to obtain a particle size of <1.0 mm. The ground biomass was stored under vacuum at room temperature and protected from moisture and light until analysis.

### 3.2. Preparation of Herbal Infusions

The initial moisture content of the OL samples was measured gravimetrically at 105 °C until constant weight was reached [88]. Based on the determined dry-matter content, the amount of fresh, air-dried, and lyophilized OL used for infusion preparation was adjusted so that each treatment represented an equivalent of 1.70 g dry matter. The OL herbal infusions were prepared according to the protocol described by Zafar et al. [18], with minor adaptations. Infusions were prepared in triplicate. An amount of fresh, air-dried, or lyophilized OL equivalent to 1.70 g dry matter was combined with 250 mL of water at 85 °C and left to infuse for 10 min. The liquid phase was then passed through Whatman^®^ Grade No. 595 filter paper (basis weight 68 g/m^2^, thickness 150 μm, pore size 4–7 μm; Whatman, Cytiva Europe GmbH, Freiburg, Germany; supplied by VWR International s.r.o., Stříbrná Skalice, Czech Republic). After filtration, the infusions were cooled to room temperature (22–25 °C) and used directly in the subsequent assays.

### 3.3. Determination of Antioxidant Capacity

Antioxidant capacity was determined directly in the OL infusions based on the free radical scavenging assay using the DPPH method. The assay was carried out according to the protocol described by Brand-Williams et al. [89], with slight modifications for the 96-well microplate as reported by Kovacikova et al. [90]. Specifically, 20 µL of each standard, blank and sample (analyzed in triplicate) were pipetted into the wells, followed by the addition of 180 µL of DPPH solution. The reaction mixture was incubated for 10 min in the dark under constant shaking at 1000 rpm. Absorbance was measured at 515 nm with a DYNARead microplate reader (Dynex Technologies s.r.o., Prague, Czech Republic). The antioxidant activity was calculated as the percentage of DPPH inhibition using the following formula:

(1) $$\%\;\mathrm{Inhibition}=\frac{A_{0}-A_{1}}{A_{0}}\times 100$$

where A_0_ represents the absorbance of the blank and A_1_ represents the absorbance of the sample. Trolox ((±)-6-hydroxy-2,5,7,8-tetramethylchroman-2-carboxylic acid; Merck KGaA, Darmstadt, Germany) diluted in ethanol (CentralChem s.r.o., Bratislava, Slovakia) served as the reference standard over a concentration range of 0–100 mg/L (R^2^ = 0.9946).

### 3.4. Determination of Total Phenolic Content

Total phenolic content was determined in the prepared OL infusions using the Folin–Ciocalteu colorimetric assay. The procedure was based on Xiong et al. [91], with slight modifications for 96-well microplates [90]. Specifically, 50 µL of distilled water was added, followed by 25 µL of the sample or standard (in triplicate), and 25 µL of Folin–Ciocalteu reagent (Merck KGaA, Darmstadt, Germany). After 6 min, 100 µL of 7.50% sodium carbonate solution (CentralChem s.r.o., Bratislava, Slovakia) was added to all wells. The plate was then left in the dark for 90 min. Absorbance was measured at 765 nm using a UV-Vis plate reader (GloMax^®^ Multi+, Promega Corp., Madison, WI, USA). Quantification was performed from a gallic acid (Merck KGaA, Darmstadt, Germany) calibration curve (0–500 mg/L; R^2^ = 0.9849) and results were expressed as mg GAE/g DW.

### 3.5. Determination of α-Amylase Inhibition Activity

α-Amylase inhibition was assessed in triplicate using a commercial α-amylase inhibitor screening kit (ab283391, Abcam Limited, Cambridge, UK) according to the manufacturer’s protocol. Briefly, the differently processed OL infusions were prepared in α-amylase assay buffer. A diluted α-amylase solution was prepared by mixing 10 µL of α-amylase enzyme with 490 µL of assay buffer. Subsequently, 50 µL of the diluted enzyme solution was added to each well containing 50 µL of test sample. The plate was incubated at room temperature for 10 min in the dark, after which 100 µL of α-amylase substrate solution was added. Absorbance at 405 nm was monitored kinetically for 24 min using a DYNARead microplate reader (Dynex Technologies s.r.o., Prague, Czech Republic). Enzyme, background, and [solvent/sample background, as appropriate] controls were included in each assay. The inhibitory effect on α-amylase was calculated as follows:

(2) $$Relative\;Inhibition\;\left(\%\right)=\frac{\left(slope\;of\;enzyme\;control-slope\;of\;sample\right)}{\left(slope\;of\;enzyme\;control\right)}\times 100$$

### 3.6. Mineral Analysis

Air-dried and lyophilized OL samples were subjected to a pre-analytical procedure using a high-performance microwave digestion system, Ethos-One (Milestone Srl, Sorisole, BG, Italy). A closed-vessel microwave-assisted acid digestion method was applied. Briefly, 5.0 mL of nitric acid (HNO_3_; ≥69%; Sigma-Aldrich Chemie GmbH, Steinheim, Germany; supplied by Lambda Life a.s., Bratislava, Slovakia) and 1.0 mL of hydrogen peroxide (H_2_O_2_; ≥30%; Sigma-Aldrich Chemie GmbH, Steinheim, Germany; supplied by Lambda Life a.s., Bratislava, Slovakia) were added to PTFE vessels. Samples and reagent blanks were digested using the manufacturer’s program for plant material. High-purity reagents suitable for trace-element analysis were used throughout to limit analytical contamination.

Element concentrations in the digested leaf samples were determined in triplicate according to the method described by Árvay et al. [92]. Each sample was analyzed using ICP-OES (Agilent ICP-OES 720, Agilent Technologies Inc., Santa Clara, CA, USA), equipped with an axial plasma configuration and an SPS-3 autosampler (Agilent Technologies GmbH, Basel, Switzerland). Calibration standards for each element were prepared to cover the expected concentration range in the analyzed samples. Operating conditions were: RF power (0.90 kW), replicate read time (3 s), instrument stabilization time (20 s), sample uptake delay (25 s), pump rate (15 rpm), rinse time (20 s), and CCD detector temperature (−35 °C). The ICP-OES gas flow parameters were set as follows: plasma (15 L/min), auxiliary (1.50 L/min) and nebulizer (1.0 L/min). The analytical wavelengths were Ag (328.068 nm), Al (167.019 nm), Ba (455.403 nm), Ca (315.887 nm), Cd (226.502 nm), Cr (267.716 nm), Cu (324.754 nm), Fe (234.350 nm), K (766.491 nm), Li (670.783 nm), Mg (383.829 nm), Mn (257.610 nm), Na (589.592 nm), Ni (231.604 nm), Pb (220.353 nm), Se (196.026 nm), Sr (407.771 nm), and Zn (206.200 nm). Multielement Standard Solution V for ICP (Sigma-Aldrich GmbH, Buchs, Switzerland) was used for calibration. Argon served as the plasma gas and carbon was used as an internal standard. Analytical accuracy was checked using the certified reference material ERM-CE278k (mussel tissue; IRMM, Geel, Belgium). The results were expressed in mg/kg DW.

### 3.7. Human Health Risk Assessment

The evaluation was based on risk indicators, including the EDI, which was calculated using the following equation:

(3) $$EDI=\frac{C\times IR}{BW}$$

where EDI represents the estimated daily intake of each element (mg/kg/BW/day), C is the average concentration of the element in air-dried or lyophilized OL (mg/g). IR is the daily ingestion rate (g/person/day), and BW is body weight. An intake of 10 g OL/person/day was adopted from the EMA/HMPC assessment report for *Olea europaea* L. [39], while the average adult BW in Europe was set at 70 kg in accordance with European Food Safety Authority (EFSA) [40]. Non-carcinogenic risk was characterized by the THQ, calculated as the ratio between EDI and the corresponding oral reference dose (RfD; mg/kg/day) [93]:

(4) $$THQ=\frac{EDI}{RfD}$$

A THQ < 1 indicates that adverse non-carcinogenic health effects are unlikely under the assumed exposure conditions, whereas a THQ > 1 may indicate a potential for adverse health risks. According to the US EPA [80], the RfDs used in this study were as follows (mg/kg/day): Ag (0.005), Al (1.0), Ba (0.20), Cu (0.04), Cr (0.003), Fe (0.70), Li (0.002), Mn (0.14), Ni (0.02), Se (0.005), Sr (0.60), and Zn (0.30). Oral reference doses for the macroelements such as Ca, Mg, Na, and K have not been established by the US EPA, as these elements are essential for normal physiological functions. Total target hazard quotient was calculated as the sum of the individual THQ values for all analyzed elements.

### 3.8. Statistical Analysis

Statistical analyses were performed using GraphPad Prism software (version 8.0.1; GraphPad Software Incorporated, San Diego, CA, USA). One-way analysis of variance (ANOVA) followed by Tukey’s multiple-comparisons test, and an unpaired *t*-test were used to determine differences among groups. Statistical significance was set at α = 0.05; results with *p* < 0.01 and *p* < 0.001 were considered highly significant and very highly significant, respectively.

## 4. Conclusions

Post-harvest processing substantially influenced the functional properties of OL herbal infusions. Among the evaluated treatments, lyophilization yielded the highest AC (15.32 ± 0.85%), TPC (65.61 ± 1.15 mg GAE/g DW), and α-amylase inhibitory activity (23.34 ± 6.08%). These findings suggest that lyophilization may improve the preservation and aqueous extractability of redox-active and enzyme-interacting constituents. Although the absence of compound-specific chromatographic analysis prevents the attribution of the observed effects to individual molecules, previous phytochemical profiling and enzyme-inhibition studies suggest that secoiridoids, particularly oleuropein and related compounds, together with flavonoids such as luteolin and its derivatives, may contribute to the antioxidant and α-amylase inhibitory responses of OL preparations [27,29,94]. The mineral profiles of air-dried and lyophilized leaves were dominated by Ca and K, while Cd and Pb remained below the analytical LOD, supporting a favorable elemental safety profile of the analyzed ‘Frantoio’ samples. Under the modeled intake assumptions, all individual THQ values and cumulative TTHQ values were below 1, indicating a low probability of adverse non-carcinogenic effects from the assessed elements. However, this risk characterization was based on total concentrations in leaves rather than on measured transfer into the prepared infusions and should therefore be interpreted specifically within the stated exposure model. Overall, lyophilization appears to be a promising post-harvest treatment for developing OL-derived infusion ingredients and for valorizing an abundant olive-production by-product. Future studies should therefore quantify individual phenolic compounds, including secoiridoids and flavonoids, using validated chromatographic and mass-spectrometric methods; investigate their bioaccessibility, metabolism, and bioavailability; and examine relationships among chemical composition, antioxidant response, and digestive-enzyme inhibition. Further studies should also include simulated gastrointestinal digestion, in vivo models, and appropriately designed human intervention studies. From a translational perspective, sensory acceptability, storage stability, technological scalability, and economic feasibility should also be considered when optimizing OL-derived ingredients for practical food and nutraceutical applications.

## Figures and Tables

**Table 1:** Relative inhibition and activity of α-amylase after treatment with OL herbal infusions.

Samples	Relative α-Amylase Inhibition (%)	Relative α-Amylase Activity (%)
Fresh OL herbal infusion	10.84 ± 2.63 ^ab^	89.16 ± 2.63 ^ab^
Air-dried OL herbal infusion	6.91 ± 1.38 ^b^	93.09 ± 1.38 ^a^
Lyophilized OL herbal infusion	23.34 ± 6.08 ^a^	76.66 ± 6.08 ^b^

OL—olive leaves; Values are presented as mean (x) ± standard deviation (SD); different lowercase letters within the same column indicate statistically significant differences among samples (One-way ANOVA with Tukey’s multiple-comparisons test; α = 0.05).

**Table 2:** Mineral profile of air-dried and lyophilized OL (mg/kg DW) analyzed using ICP-OES.

Elements	Air-Dried OL	Lyophilized OL	*p*-Value
Macroelements			
K	7467.24 ± 14.58 ^a^	6448.58 ± 11.42 ^b^	*p* < 0.001
Ca	6054.29 ± 20.57 ^b^	6931.89 ± 21.52 ^a^	*p* < 0.001
Na	138.55 ± 0.52 ^a^	120.56 ± 0.51 ^b^	*p* < 0.001
Mg	72.62 ± 0.08 ^b^	76.04 ± 0.16 ^a^	*p* < 0.001
Trace elements			
Al	65.53 ± 0.94 ^b^	86.43 ± 1.03 ^a^	*p* < 0.001
Sr	23.50 ± 0.00 ^b^	27.39 ± 0.02 ^a^	*p* < 0.001
Fe	21.54 ± 0.16 ^b^	23.58 ± 0.13 ^a^	*p* < 0.001
Mn	11.88 ± 0.02 ^a^	11.58 ± 0.01 ^b^	*p* < 0.001
Zn	3.79 ± 0.12 ^b^	4.36 ± 0.25 ^a^	*p* < 0.05
Ba	3.06 ± 0.01 ^b^	3.35 ± 0.01 ^a^	*p* < 0.001
Cu	2.43 ± 0.01 ^a^	1.80 ± 0.00 ^b^	*p* < 0.001
Se	1.30 ± 0.33 ^a^	0.09 ± 0.00 ^b^	*p* < 0.01
Ni	0.35 ± 0.15 ^b^	1.18 ± 0.03 ^a^	*p* < 0.001
Cr	0.15 ± 0.01	0.13 ± 0.02	ns
Li	0.04 ± 0.00 ^b^	0.05 ± 0.00 ^a^	*p* < 0.001
Ag	0.01 ± 0.00	0.02 ± 0.00	ns
Cd, Pb	<LOD ^1^	<LOD ^1^	-

OL—olive leaves; ^1^ <LOD—below the limit of detection; ns—not significant; Values are presented as mean (x) ± standard deviation (SD); different lowercase letters indicate statistically significant differences among samples (unpaired *t*-test; α = 0.05).

**Table 3:** Estimated daily intake (EDI) of elements for adults due to the consumption of OL (mg/kg BW/day).

Elements	Estimated Daily Intake (EDI)	
	Air-Dried OL	Lyophilized OL
K	1.066748	0.921225
Ca	0.864898	0.990270
Na	0.019792	0.017222
Mg	0.010374	0.010862
Al	0.009361	0.012347
Sr	0.003357	0.003912
Fe	0.003077	0.003368
Mn	0.001697	0.001654
Zn	0.000541	0.000622
Ba	0.000437	0.000478
Cu	0.000347	0.000257
Se	0.000185	0.000012
Ni	0.000050	0.000168
Cr	0.000021	0.000018
Li	0.000005	0.000007
Ag	0.000001	0.000002

OL—olive leaves.

**Table 4:** Target hazard quotient (THQ) and total target hazard quotient (TTHQ) for elements in air-dried and lyophilized OL.

Elements	Samples	
	Air-Dried OL	Lyophilized OL
Target hazard quotient (THQ)		
Se	0.037000	0.002400
Mn	0.012121	0.011814
Al	0.009361	0.012347
Cu	0.008675	0.006425
Cr	0.007000	0.006000
Sr	0.005595	0.006520
Fe	0.004396	0.004811
Li	0.002500	0.003500
Ni	0.002500	0.008400
Ba	0.002185	0.002390
Zn	0.001803	0.002073
Ag	0.000200	0.000400
Total target hazard quotient (TTHQ)		
	0.09336	0.067081

OL—olive leaves.

## Data Availability

The original contributions presented in this study are included in the article. Further inquiries can be directed to the corresponding authors.

## Abbreviations

The following abbreviations are used in this manuscript:

OL	Olive leaves
AC	Antioxidant capacity
DPPH	2,2-diphenyl-1-picrylhydrazyl
TPC	Total phenolic content
ICP-OES	Inductively coupled plasma optical emission spectrometry
EDI	Estimated daily intake
THQ	Target hazard quotient
TTHQ	Total target hazard quotient
GAE	Gallic acid equivalent
DW	Dry weight
Ca	Calcium
K	Potassium
Cd	Cadmium
Pb	Lead
Na	Sodium
Mg	Magnesium
P	Phosphorus
Mn	Manganese
Fe	Iron
Cu	Copper
Zn	Zinc
ANOVA	One-way analysis of variance
SD	Standard deviation
HbA1c	Hemoglobin A1c
IRI	Immunoreactive insulin
FPG	Fasting plasma glucose
LOD	Limit of detection
ns	Not significant
Al	Aluminum
Sr	Strontium
Ba	Barium
Se	Selenium
Ni	Nickel
Cr	Chromium
Li	Lithium
Ag	Silver
S	Sulfur
V	Vanadium
US EPA	United States Environmental Protection Agency
RfD	Oral reference dose
BW	Body weight
BBCH	Biologische Bundesanstalt Bundessortenamt and Chemische Industrie
EFSA	European Food Safety Authority

## References

[1] Schwingshackl L., Schwedhelm C., Galbete C., Hoffmann G. (2017). Adherence to Mediterranean Diet and Risk of Cancer: An Updated Systematic Review and Meta-Analysis. Nutrients.

[2] Farias-Pereira R., Zuk J.B., Khavaran H. (2023). Plant bioactive compounds from Mediterranean diet improve risk factors for metabolic syndrome. Int. J. Food Sci. Nutr..

[3] Capurso A., Crepaldi G., Capurso C. (2020). The Mediterranean Diet: A Pathway to Successful Aging. Aging Clin. Exp. Res..

[4] Obeid C.A., Gubbels J.S., Jaalouk D., Kremers S.P.J., Oenema A. (2022). Adherence to the Mediterranean Diet among Adults in Mediterranean Countries: A Systematic Literature Review. Eur. J. Nutr..

[5] Besnard G., Khadari B., de Navascués M., Fernández-Mazuecos M., El Bakkali A., Arrigo N., Baali-Cherif D., Bronzini de Caraffa V., Santoni S., Vargas P. (2013). The complex history of the olive tree: From Late Quaternary diversification of Mediterranean lineages to primary domestication in the northern Levant. Proc. R. Soc. B.

[6] Uylaşer V., Yildiz G. (2014). The historical development and nutritional importance of olive and olive oil constituted an important part of the Mediterranean diet. Crit. Rev. Food Sci. Nutr..

[7] Fraga H., Moriondo M., Leolini L., Santos J.A. (2021). Mediterranean olive orchards under climate change: A review of future impacts and adaptation strategies. Agronomy.

[8] Tabera J., Guinda Á., Ruiz-Rodríguez A., Señoráns F.J., Ibáñez E., Albi T., Reglero G. (2004). Countercurrent supercritical fluid extraction and fractionation of high-added-value compounds from a hexane extract of olive leaves. J. Agr. Food Chem..

[9] Filgueira-Garro I., González-Ferrero C., Mendiola D., Marín-Arroyo M.R. (2022). Effect of cultivar and drying methods on phenolic compounds and antioxidant capacity in olive (*Olea europaea* L.) leaves. AIMS Agric. Food.

[10] Palmeri R., Siracusa L., Carrubba M., Parafati L., Proetto I., Pesce F., Fallico B. (2022). Olive Leaves, a Promising Byproduct of Olive Oil Industry: Assessment of Metabolic Profiles and Antioxidant Capacity as a Function of Cultivar and Seasonal Change. Agronomy.

[11] Markhali F.S., Teixeira J.A., Rocha C.M.R. (2020). Olive Tree Leaves-A Source of Valuable Active Compounds. Processes.

[12] Clodoveo M.L., Crupi P., Annunziato A., Corbo F. (2022). Innovative Extraction Technologies for Development of Functional Ingredients Based on Polyphenols from Olive Leaves. Foods.

[13] Markhali F.S., Teixeira J.A. (2024). Extractability of oleuropein, hydroxytyrosol, tyrosol, verbascoside and flavonoid-derivatives from olive leaves using ohmic heating: A green process for value addition. Sustain. Food Technol..

[14] Benavente-García O., Castillo J., Lorente J., Ortuno A., Del Rio J.A. (2000). Antioxidant activity of phenolics extracted from *Olea europaea* L. leaves. Food Chem..

[15] Perrinjaquet-Moccetti T., Busjahn A., Schmidlin C., Schmidt A., Bradl B., Aydogan C. (2008). Food supplementation with an olive (*Olea europaea* L.) leaf extract reduces blood pressure in borderline hypertensive monozygotic twins. Phytother. Res..

[16] Monteleone J.I., Sperlinga E., Siracusa L., Spagna G., Parafati L., Todaro A., Palmeri R. (2021). Water as a Solvent of Election for Obtaining Oleuropein-Rich Extracts from Olive (*Olea europaea*) Leaves. Agronomy.

[17] Ramírez E.M., Brenes M., Romero C., Medina E. (2023). Olive leaf processing for infusion purposes. Foods.

[18] Zafar A., Ahmed W., Tahir S.K., Ahmad A.M.R., Iqbal S. (2025). Therapeutic effects of olive leaf tea (*Olea europaea* L.) on gastrointestinal symptoms and body composition in adults with small intestinal bacterial overgrowth. Front. Nutr..

[19] Preczenhak A.P., Sartori J.A.d.S., Fortes C.M.H., Kluge R.A., Bertoncini E.I., Prati P. (2025). Influence of the preparation method on the bioactive compounds of olive tree leaf tea. Braz. J. Food Technol..

[20] Meireles M., Cortez-Ribeiro A.C., Polck D., Almeida-de-Souza J., Ferro-Lebres V. (2023). Olive Leaf Tea Impact on Postprandial Glycemia: A Randomized Cross-Over Trial. Foods.

[21] De Leonardis A., Macciola V., Cuomo F., Lopez F. (2015). Evidence of oleuropein degradation by olive leaf protein extract. Food Chem..

[22] Bhatta S., Stevanovic Janezic T., Ratti C. (2020). Freeze-Drying of Plant-Based Foods. Foods.

[23] Afaneh I., Yateem H., Al-Rimawi F. (2015). Effect of olive leaves drying on the content of oleuropein. Am. J. Anal. Chem..

[24] Kamran M., Hamlin A.S., Scott C.J., Obied H.K. (2015). Drying at high temperature for a short time maximizes the recovery of olive leaf biophenols. Ind. Crops Prod..

[25] Ahmad-Qasem M.H., Ahmad-Qasem B.H., Barrajón-Catalán E., Micol V., Cárcel J.A., García-Pérez J.V. (2016). Drying and storage of olive leaf extracts. Influence on polyphenols stability. Ind. Crops Prod..

[26] Feng S., Zhang C., Liu L., Xu Z., Chen T., Zhou L., Yuan M., Li T., Ding C. (2021). Comparison of Phenolic Compounds in Olive Leaves by Different Drying and Extraction Methods. Separations.

[27] Zhang C., Zhang J., Xin X., Zhu S., Niu E., Wu Q., Li T., Liu D. (2022). Changes in Phytochemical Profiles and Biological Activity of Olive Leaves Treated by Two Drying Methods. Front. Nutr..

[28] Ćorković I., Gašo-Sokač D., Pichler A., Šimunović J., Kopjar M. (2022). Dietary Polyphenols as Natural Inhibitors of α-Amylase and α-Glucosidase. Life.

[29] Komaki E., Yamaguchi S., Maru I., Kinoshita M., Kakehi K., Ohta Y., Tsukada Y. (2003). Identification of Anti-α-Amylase Components from Olive Leaf Extracts. Food Sci. Technol. Res..

[30] Abaza L., Taamalli W., Temine S.B., Daound D., Gutierrez F., Zarrouk M. (2005). Natural antioxidant composition as correlated to stability of some Tunisian virgin olive oils. Riv. Ital. Delle Sostanze Grasse.

[31] Özcan M.M., Fındık S., AlJuhaimi F., Ghafoor K., Babiker E.E., Adiamo O.Q. (2019). The effect of harvest time and varieties on total phenolics, antioxidant activity and phenolic compounds of olive fruit and leaves. J. Food Sci. Technol..

[32] Pasković I., Franić M., Chatzistathis T., Pongrac P., Žurga P., Germek V.M., Palčić I., Goreta Ban S., Zakraoui M., Marcelić Š. (2025). Optimising Olive Leaf Phenolic Compounds: Cultivar and Temporal Interactions. Plants.

[33] De Oliveira N.M., Lopes L., Chéu M.H., Soares E., Meireles D., Machado J. (2023). Updated Mineral Composition and Potential Therapeutic Properties of Different Varieties of Olive Leaves from *Olea europaea*. Plants.

[34] Pashtetskaia A., Kravtsova A., Peshkova A., Zinicovscaia I., Shevchuk O. (2025). Mineral Composition of *Olea europaea* L. Leaves and Tisanes. Plants.

[35] Bahloul N., Kechaou N., Mihoubi N.B. (2014). Comparative investigation of minerals, chlorophylls contents, fatty acid composition and thermal profiles of olive leaves (*Olea europeae* L.) as by-product. Grasas Aceites.

[36] Terker A.S., Zhang C., McCormick J.A., Lazelle R.A., Zhang C., Meermeier N.P., Siler D.A., Park H.J., Fu Y., Cohen D.M. (2015). Potassium modulates electrolyte balance and blood pressure through effects on distal cell voltage and chloride. Cell Metab..

[37] Terwoord J.D., Hearon C.M., Luckasen G.J., Richards J.C., Joyner M.J., Dinenno F.A. (2018). Elevated extracellular potassium prior to muscle contraction reduces onset and steady-state exercise hyperemia in humans. J. Appl. Physiol..

[38] McCarron D.A., Reusser M.E. (2001). Are low intakes of calcium and potassium important causes of cardiovascular disease?. Am. J. Hypertens..

[39] European Medicines Agency, Committee on Herbal Medicinal Products (EMA/HMPC) (2017). Assessment Report on Olea europaea L., Folium.

[40] European Food Safety Authority (EFSA) Scientific Committee (2012). Guidance on selected default values to be used by the EFSA Scientific Committee, Scientific Panels and Units in the absence of actual measured data. EFSA J..

[41] Şahin S., Bilgin M. (2018). Olive tree (*Olea europaea* L.) leaf as a waste by-product of table olive and olive oil industry: A review. J. Sci. Food Agric..

[42] Ramírez E., Medina E., Brenes M., Romero C. (2014). Endogenous enzymes involved in the transformation of oleuropein in Spanish table olive varieties. J. Agric. Food Chem..

[43] Delgado-Povedano M.d.M., Priego-Capote F., Luque de Castro M.D. (2017). Selective ultrasound-enhanced enzymatic hydrolysis of oleuropein to its aglycon in olive (*Olea europaea* L.) leaf extracts. Food Chem..

[44] Şahin S., Ahmed Malik N.S., Perez J.L., Brockington J.E. (2012). Seasonal changes of individual phenolic compounds in leaves of twenty olive cultivars grown in Texas. J. Agric. Sci. Technol..

[45] Brahmi F., Mechri B., Dabbou S., Dhibi M., Hammami M. (2012). The efficacy of phenolic compounds with different polarities as antioxidants from olive leaves depending on seasonal variations. Ind. Crops Prod..

[46] Brahmi F., Mechri B., Dhibi M., Hammami M. (2015). Effect of growth stage and solvent extract on the antioxidant potential of olive leaves. J. Plant Sci..

[47] Talhaoui N., Taamalli A., Gómez-Caravaca A.M., Fernández-Gutiérrez A., Segura-Carretero A. (2015). Phenolic compounds in olive leaves: Analytical determination, biotic and abiotic influence, and health benefits. Food Res. Int..

[48] Talhaoui N., Gómez-Caravaca A.M., Roldán C., León L., De la Rosa R., Fernández-Gutiérrez A., Segura-Carretero A. (2015). Chemometric analysis for the evaluation of phenolic patterns in olive leaves from six cultivars at different growth stages. J. Agric. Food Chem..

[49] Blasi F., Urbani E., Simonetti M.S., Chiesi C., Cossignani L. (2016). Seasonal variations in antioxidant compounds of *Olea europaea* leaves collected from different Italian cultivars. J. Appl. Bot. Food Qual..

[50] Orak H.H., Isbilir S.S., Yagar H. (2012). Determination of antioxidant properties of lyophilized olive leaf water extracts obtained from 21 different cultivars. Food Sci. Biotechnol..

[51] Yıldırım A., Mavi A., Oktay M., Kara A.A., Algur Ö.F., Bilaloǧlu V. (2000). Comparison of antioxidant and antimicrobial activities of tilia (*Tilia argentea Desf Ex DC*), sage (*Salvia Triloba* L.), and black tea (*Camellia sinensis*) extracts. J. Agric. Food Chem..

[52] Kähkönen M.P., Hopia A.I., Heinonen M. (2001). Berry Phenolics and Their Antioxidant Activity. J. Agric. Food Chem..

[53] Hnatová L., Kováčiková E., Gálik B., Novotná I., Jančo I., Kopčeková J., Fatrcová Šramková K., Lenická D., Lenický M., Mlyneková E. (2026). Bioactive and nutritional potential of extracts from differently processed olive leaves (*Olea europaea* L.) as by-products. J. Cent. Eur. Agric..

[54] Castillo-Luna A., Miho H., Ledesma-Escobar C.A., Priego-Capote F. (2023). Comparison of Drying Techniques for Extraction of Bioactive Compounds from Olive-Tree Materials. Foods.

[55] Ahmad-Qasem M.H., Barrajón-Catalán E., Micol V., Mulet A., García-Pérez J.V. (2013). Influence of freezing and dehydration of olive leaves (var. Serrana) on extract composition and antioxidant potential. Food Res. Int..

[56] Chaji S., Zenasni W., Ouaabou R., Ajal E.A., Lahlali R., Fauconnier M.-L., Hanine H., Černe M., Pasković I., Merah O. (2024). Nutrient and Bioactive Fraction Content of *Olea europaea* L. Leaves: Assessing the Impact of Drying Methods in a Comprehensive Study of Prominent Cultivars in Morocco. Plants.

[57] Narra F., Piragine E., Benedetti G., Ceccanti C., Florio M., Spezzini J., Troisi F., Giovannoini R., Martelli A., Guidi L. (2024). Impact of thermal processing on polyphenols, carotenoids, glucosinolates, and ascorbic acid in fruit and vegetables and their cardiovascular benefits. Compr. Rev. Food Sci. Food Saf..

[58] Karam M.C., Petit J., Zimmer D., Djantou E.B., Scher J. (2016). Effects of Drying and Grinding in Production of Fruit and Vegetable Powders: A Review. J. Food Eng..

[59] Zoric Z., Dragovi V., Pedisi S., Garofuli I.E. (2014). Kinetics of the degradation of anthocyanins, phenolic acids and flavonols during heat treatments of freeze-dried sour cherry marasca paste. Food Technol. Biotechnol..

[60] Nowak D., Jakubczyk E. (2020). The Freeze-Drying of Foods-The Characteristic of the Process Course and the Effect of Its Parameters on the Physical Properties of Food Materials. Foods.

[61] Pourghorban S., Yadegarian L., Jalili M., Rashidi L. (2022). Comparative Physicochemical, Microbiological, Antioxidant, and Sensory properties of pre- and post-fermented yoghurt enriched with olive leaf and its extract. Food Sci. Nutr..

[62] Aydın B.D., Konak Göktepe Ç., Akın N. (2025). Enhancing Set-Type Yoghurt with Olive Leaf Extract: A Comparison of Powdered Addition and Surface Spraying on Quality, Functionality, and Sensory Attributes. Food Sci. Nutr..

[63] Potsaki P., Papagianni O.I., Almpounioti K., Soulakellis C., Voutsa A., Katira O., Bountziouka V., Karantonis C., Koutelidakis A.E. (2025). Acute Effects of Olive Leaf Tea and Olive Leaf Powder Biscuits on Postprandial Glycemia, Lipid Profile and Inflammatory Markers: A Randomized Controlled Crossover Trial in Healthy Volunteers. Appl. Sci..

[64] Šarolić M., Milišić E., Sinovčić D., Crnjac A., Brzović P., Vukušić B., Radman S. (2026). Application of Freeze-Dried Olive Leaf Powder in Cracker Formulation: Effects on Phenolics, Antioxidant Activity, Volatile Profile, and Sensory Quality. Processes.

[65] Tripodi G., Torre M., Verzera A., Cincotta F. (2026). Influence of Drying Methods on Volatile Aroma Compounds and Sensory Properties of Olive Leaf Herbal Tea. Foods.

[66] Vieitez I., Ellis A.C., Aldecosea T., Dauber C., Irigaray B., Baez J., Gámbaro A. (2026). Influence of Extraction Conditions on the Sensory Profile, Acceptability, and Polyphenol Content of Infusions from Arbequina Olive Leaves. Food Humanit..

[67] McDougall G.J., Shpiro F., Dobson P., Smith P., Blake A., Stewart D. (2005). Different polyphenolic components of soft fruits inhibit alpha-amylase and alpha-glucosidase. J. Agric. Food Chem..

[68] Al-Dabbas M.M., Al-Jaloudi R., Hamad H.J., Al-Nawasrah B.A., Al-Refaie D., Abughoush M., Aldabbas A., Iqbal S. (2025). Antioxidant, Antibacterial, and Antidiabetic Activities of Different Extracts from Wild Olive Leaves Grown in United Arab Emirates. Processes.

[69] Hammoudi R., Dehak K., Tlili M.L., Khenfer S., Medjouel M., Hadj-Mahammed M. (2017). Biological Activities of Phenolic Extracts of a Medicinal Plant, Endemic to the Algerian Sahara: Salvia Chudaei Batt. & Trab. Int. J. Biosci..

[70] Rasouli H., Hosseini-Ghazvini S.M.B., Adibi H., Khodarahmi R. (2017). Differential α-Amylase/α-Glucosidase Inhibitory Activities of Plant-Derived Phenolic Compounds: A Virtual Screening Perspective for the Treatment of Obesity and Diabetes. Food Funct..

[71] Striegel L., Kang B., Pilkenton S.J., Rychlik M., Apostolidis E. (2015). Effect of Black Tea and Black Tea Pomace Polyphenols on α-Glucosidase and α-Amylase Inhibition, Relevant to Type 2 Diabetes Prevention. Front. Nutr..

[72] Yilmazer-Musa M., Griffith A.M., Michels A.J., Schneider E., Frei B. (2012). Grape Seed and Tea Extracts and Catechin 3-Gallates Are Potent Inhibitors of α-Amylase and α-Glucosidase Activity. J. Agric. Food Chem..

[73] Wainstein J., Ganz T., Boaz M., Dayan Y.B., Dolev E., Kerem Z., Madar Z. (2012). Olive Leaf Extract as a Hypoglycemic Agent in Both Human Diabetic Subjects and in Rats. J. Med. Food.

[74] Araki R., Fujie K., Yuine N., Watabe Y., Nakata Y., Suzuki H., Isoda H., Hashimoto K. (2019). Olive leaf tea is beneficial for lipid metabolism in adults with prediabetes: An exploratory randomized controlled trial. Nutr. Res..

[75] Araki R., Fujie K., Nakata Y., Suzuki H., Matsui K., Uematsu K., Shibasaki H., Ando T., Ueyama Y., Isoda H. (2018). An exploratory study of the effects of continuous intake of olive leaf tea on physique and glucose and lipid metabolism. J. Jpn. Soc. Nutr. Food Sci..

[76] Nikou T., Sakavitsi M.E., Kalampokis E., Halabalaki M. (2022). Metabolism and Bioavailability of Olive Bioactive Constituents Based on In Vitro, In Vivo and Human Studies. Nutrients.

[77] Jordán M., García-Acosta N., Espartero J.L., Goya L., Mateos R. (2025). Hydroxytyrosol Bioavailability: Unraveling Influencing Factors and Optimization Strategies for Dietary Supplements. Nutrients.

[78] Khalil A.A., Rahman M.M., Rauf A., Islam M.R., Manna S.J., Khan A.A., Ullah S., Akhtar M.N., Aljohani A.S.M., Abdulmonem W.A. (2024). Oleuropein: Chemistry, extraction techniques and nutraceutical perspectives-An update. Crit. Rev. Food Sci. Nutr..

[79] Antunes B.d.F., Otero D.M., Bonemann D.H., Ribeiro A.S., Jacques A.C., Zambiazi R.C. (2021). Evaluation of physicochemical, bioactive composition and profile of fatty acids in leaves of different olive cultivars. Rev. Ceres.

[80] Gagour J., Ibourki M., Antari A.E., Dakar E.H., Aissa R., Giuffrè A.M., Laknifli A., Gharby S. (2024). Leaf mineral profiling and its correlation with oil physicochemical traits from four olive (*Olea europaea* L.) cultivars grown in Morocco as affected by olive ripening stages. Eur. Food Res. Technol..

[81] Tunç Y., Yaman M., Keçe Y.M., Yilmaz K.U., Yildiz E., Güneş A. (2025). Characterization of olive (*Olea europaea* L.) cultivars; colour properties, biochemical contents, antioxidant activity and nutrient contents. Genet. Resour. Crop Evol..

[82] Pucci E., Palumbo D., Puiu A., Lai A., Fiorani L., Zoani C. (2022). Characterization and Discrimination of Italian Olive (*Olea europaea sativa*) Cultivars by Production Area Using Different Analytical Methods Combined with Chemometric Analysis. Foods.

[83] Cavalheiro C.V., Picoloto R.S., Cichoski A.J., Wagner R., de Menezes C.R., Zepka L.Q., Da Croce D.M., Barin J.S. (2015). Olive leaves offer more than phenolic compounds—Fatty acids and mineral composition of varieties from Southern Brazil. Ind. Crops Prod..

[84] Ozyigit I.I., Karahan F., Yalcin I.E., Hocaoglu-Ozyigit A., Ilcim A. (2022). Heavy metals and trace elements detected in the leaves of medicinal plants collected in the southeast part of Turkey. Arab. J. Geosci..

[85] Tefera M., Demissie G., Guadie A., Lakew W., Sewachen B., Ayalew M., Shumye D. (2025). Heavy metals pollution in cancer and wound-healing medicinal plants: A comprehensive assessment of health risk. Food Chem. Adv..

[86] United States Environmental Protection Agency (US EPA) (2024). Regional Screening Level (RSL) Subchronic Toxicity Supporting Table, November 2024.

[87] Brima E.I., Siddeeg S.M. (2022). Pilot Study of Trace Elements in the Infusion of Medicinal Plants Used for Diabetes Treatment. Int. J. Anal. Chem..

[88] AOAC (1997). Official Methods of Analysis of AOAC International.

[89] Brand-Williams W., Cuvelier M.E., Berset C. (1995). Use of a free radical method to evaluate antioxidant activity. LWT.

[90] Kovacikova E., Urbanova L., Kuzelova L., Lakatosova J., Janco I., Arvay J., Ziarovska J., Duranova H. (2025). Chemical-analytical characterization and Pun1 gene expression variability analysis of ten selected *Capsicum* varieties. Food Biosci..

[91] Xiong J., Chan Y.H., Rathinasabapathy T., Grace M.H., Komarnytsky S., Lila M.A. (2020). Enhanced stability of berry pomace polyphenols delivered in protein-polyphenol aggregate particles to an in vitro gastrointestinal digestion model. Food Chem..

[92] Árvay J., Šnirc M., Hauptvogl M., Bilčíková J., Bobková A., Demková L., Hudáček M., Hrstková M., Lošák T., Král M. (2019). Concentration of micro- and macro-elements in green and roasted coffee: Influence of roasting degree and risk assessment for the consumers. Biol. Trace Elem. Res..

[93] Kovacik A., Helczman M., Arvay J., Tomka M., Snirc M., Janco I., Fik M., Stefunkova N., Dupak R., Harangozo L. (2025). Microelements, fatty acid profile, and selected biomarkers in grass carp (*Ctenopharyngodon idella*) muscle tissue: Seasonal variations and health risk assessment. Biol. Trace Elem. Res..

[94] Hadrich F., Bouallagui Z., Junkyu H., Isoda H., Sayadi S. (2015). The α-Glucosidase and α-Amylase Enzyme Inhibitory of Hydroxytyrosol and Oleuropein. J. Oleo Sci..

